# Pregnenolone Bioproduction in Engineered Methylobacteria: Design and Elaboration

**DOI:** 10.3390/ijms262210975

**Published:** 2025-11-13

**Authors:** Daria Tekucheva, Veronika Poshekhontseva, Dmitry Fedorov, Mikhail Karpov, Ludmila Novikova, Alexey Zamalutdinov, Marina Donova

**Affiliations:** 1 G.K. Skryabin Institute of Biochemistry and Physiology of Microorganisms (IBPM), Russian Academy of Sciences, Federal Research Center “Pushchino Scientific Center for Biological Research of the Russian Academy of Sciences”, Prospekt Nauki 5, Pushchino, Moscow 142290, Russia; rikahameleon@mail.ru (V.P.);; 2 Belozersky Institute of Physico-Chemical Biology, M.V. Lomonosov Moscow State University, Leninskie Gory 1/40, Moscow 119991, Russia

**Keywords:** cytochrome P450scc (CYP11A1), cholesterol, pregnenolone, heterologous expression, methylobacteria, *Methylorubrum extorquens*

## Abstract

In this study, for the first time, the genes encoding the mammalian steroidogenesis system—cytochrome P450scc (CYP11A1), and its native redox partners adrenodoxin and adrenodoxin reductase—were successfully expressed in the methylobacterium *Methylorubrum extorquens*. The advantage of using methylobacteria as an expression chassis is that they grow on inexpensive mineral media, use methanol as a carbon and energy source, and do not possess their own sterol catabolism systems. Using recombinant methylobacteria, the valuable steroid pregnenolone was obtained as a sole metabolite from cholesterol. The effect of media composition, bioconversion conditions such as methanol and N-sources content, modes of substrate addition, detergents, methyl-β-cyclodextrin, biomass, and aeration on pregnenolone accumulation was investigated. Under optimized conditions, its yield exceeded 100 mg/L. The results demonstrate a proof of concept relating to the use of bacteria lacking their own steroid degradation systems as microbial chassis for heterologous steroidogenesis systems, including mammalian cytochrome CYP11A1.

## 1. Introduction

Steroids are natural regulators of vital processes, with high biological activity and wide therapeutic applications. By their chemical nature, steroids represent the gonane core, with differences in the location and stereochemistry of the functional groups attached to it.

Pregnenolone (pregn-5-en-3β-ol-20-one) is a neurosteroid with pleiotropic effects that regulates descending brain functions as a neuromodulator and neurotransmitter. In mammals, pregnenolone is synthesized primarily in the gonads, placenta, and adrenal cortex, as well as in the cells of the nervous system and heart [1]. Pregnenolone accumulation is the first, rate-limiting, and hormonally regulated step of mammal’s steroidogenesis.

Pregnenolone is a key precursor to a number of steroid hormones, including androgens, estrogens, glucocorticoids, mineralocorticoids, and progestogens that are vital to mammals and are used in the production of many drugs.

Pregnenolone plays an important role in the learning and memory [2], and its level in the brain decreases with age, in bipolar disorder, or, in contrast, is elevated in patients with Alzheimer’s disease [3]. Pregnenolone’s neuroprotective function has been demonstrated in acute and chronic stress, anxiety, depression, psychosis disorders, and in cannabinoid-related dysfunctions [4]. New strategies for the treatment of certain types of cancer [5], neurodegenerative diseases, and spinal cord injuries [4,6], as well as viral infections [7] and leishmaniasis [8] include the use of pregnenolone or its derivatives.

The cholesterol hydroxylase/C20-C22 lyase (CH/L) system, responsible for the conversion of cholesterol to pregnenolone, consists of cytochrome P450scc (CYP11A1) and its Red/Ox partners. CYP11A1 is the product of a single gene, the cDNA of which has been firstly isolated from bovine adrenal cortex mRNA [9] and then cloned from different species, including mice and humans [1].

In the inner mitochondrial membrane, CYP11A1 catalyzes the hydroxylation of cholesterol at C22 and C20 positions to form the intermediates 22(R)-hydroxycholesterol and 20α,22-dihydroxycholesterol, followed by cleavage of the cholesterol side chain resulting in pregnenolone and isocaproaldehyde [1]. During the reaction, flavoprotein adrenodoxin reductase (AdR) donates an electron from NADPH to iron–sulfur protein adrenodoxin (Adx), which transfers the electron to the heme group of CYP11A1 in the mitochondrial matrix [10].

The microbiological production of steroid drugs has great potential due to its environmental safety and, in addition, its sustainability. In comparison with chemical and semi-synthetic methods, microbial catalysis often provides a reduction in the overall production scheme and higher regio- and stereospecificity of the reactions. The microbial biocatalysis of steroids using microorganisms genetically edited by expressing heterologous mammalian proteins is currently of great interest.

CYP11A1 was first heterologically synthesized in *Escherichia coli*, which lack their own cytochromes P450, but the target activity of membrane fractions containing recombinant CYP11A1 was possible only when the native redox partners Adx and AdR were added [11]. The tricistronic genetic construct that included cDNA sequences of AdR_Adx_P450scc divided by ribosome binding sites was created as described in [12], and such an order of heterologous cDNAs in the plasmid expression cassette was recognized as optimal, based on the synthetized enzymes stoichiometric ratio [13]. The resulting *E. coli* strains carrying such a construct were able to transform cholesterol to pregnenolone with the obtained titer of 0.42–0.62 mg/L [12,13].

The idea of the self-sufficient biosynthesis of pregnenolone and then progesterone from the endogenous steroid substrate was developed in *Saccharomyces cerevisiae* expressing all three components of the mammalian CH/L system by [14]. However, the yeast endogenous sterols, such as ergosterol and brassicasterol, contain a C20–C22 double bond in the side chain and are, therefore, not suitable substrates for CYP11A1. To solve the problem, additional genetic modifications had been performed—including the integration of Δ^7^-reductase from *Arabidopsis thaliana* and type II human 3β-steroid dehydrogenase, and the deletion of Δ^22^-desaturase. A pregnenolone titer reached 60 mg/L within 100 h of the cultivation of the engineered yeast on the galactose medium. A pregnenolone titer of 78 mg/L was obtained in a 5 l bioreactor with a campesterol-producing strain of *Yarrowia lipolytica*, which has an advantage over *S. cerevisiae* in the expression of mammalian proteins, its high level of native acetyl-CoA, and the ability to accumulate lipid bodies as a reserve nutrient [15].

The use of actinobacteria of the genus *Mycolicibacterium* as an expression chassis for the production of pregnenolone and progesterone was reported. However, *Mycolicibacterium smegmatis* and related mycolicibacteria have their own multifunctional catalytic systems for steroid oxidation. The reconstruction of the eukaryotic CH/L system in mycolicibacteria led to the conversion of sterols into pregnenolone, which, in turn, was oxidized to progesterone and subsequently destroyed by the intrinsic host enzymes [16]. To overcome this bottleneck, 3-methoxymethyl esters of chole- and phytosterols were synthesized, and were transformed into 3-methoxymethylpregnenolone within 48 h, with an efficiency of 85.2%. The subsequent removal of the chemical protection (methoxymethyl group) allowed for the almost equimolar regeneration of the 3β-OH group to yield pregnenolone [17]. Therefore, the additional chemical stages were required.

Pregnenolone formation from chole-, sito-, and campesterols was demonstrated for the soil bacterium *Bacillus megaterium* MS941, which heterologously expressed genes encoding CYP11A1 and its native redox partners under the control of an inducible strong xylose promoter [18]. *B. megaterium* is well-known for its ability to accumulate poly-3β-hydroxybutyrate (PHB) granules for energy and carbon storage in the cytosol. Such dranules are coated with a monophospholipid membrane rich in PHB metabolizing enzymes [19]. CYP11A1 and AdR have been shown to be embedded in the lipid monolayer of PHB granules, and cholesterol can be accumulated in the core of PHB granules. Indeed, the recombinant *B. megaterium* strain lacking PHB polymerase was unable to form granules, and showed reduced pregnenolone yield [18].

In this study, we proposed to use *Methylorubrum extorquens* as a chassis for the the heterologous expression of the genes encoding the cholesterol hydroxylase/C20–C22 lyase (CHL) enzyme system for several reasons.

Firstly, *M. extorquens* AM1 is a type II methanotroph related to *Alphaproteobacteria,* and is capable of utilizing C1 carbon sources (methanol, methylamine, and formate) for metabolism via the serine cycle or for energy production via formate dehydrogenases, as well as multicarbon compounds for biomass accumulation [20,21,22]. For over 50 years, *M. extorquens* AM1 has served as the main model system for studying the so-called methylotrophy, i.e., the growth on C1 substrates [23,24]. Thus, an important advantage of using *M. extorquens* AM1 as a potential expression chassis is the ability to grow in a simple methanol medium, which is inexpensive, renewable, does not compete with food industry, and reduces the risk of biocontamination [25].

The second reason is the extensive knowledge of genetics and metabolism of *M. extorquens* AM1 [24,26,27,28]. Over the past decades, a large number of genetic tools have been developed for *M. extorquens* [29,30,31]. This has allowed the creation of genetically modified strains that are used in the bioproduction of high-value products, such as mesaconic, methylsuccinic [32], and itaconic acids [25], butanol-1 [33], copolymers of polyhydroxyalkanoates [34], and others.

Thirdly, *M. extorquens* AM1 is able to accumulate PHB granules that are up to 42% of cell biomass [35], which provided an optimal environment for the functional association of the proteins CYP11A1 and AdR and for the accumulation of poorly soluble sterols, as shown by Gerber et al. [18], using recombinant *B. megaterium*.

And finally, of particular importance, is the absence in the methylobacteria from their own sterol catabolism system, which can facilitate the selective accumulation of the target product and prevent undesirable steroid core degradation due to the functioning of only the heterologous steroidogenesis system.

We investigated the possibility of using methylobacteria as an expression chassis for the heterologous expression of the genes encoding cytochrome P450scc (CYP11A1) and its native partners, AdR and Adx, of the bovine adrenal cortex.

## 2. Results

### 2.1. Creation of Recombinant Strains of M. Extorquens AM1 Providing Bioconversion of Cholesterol to Pregnenolone

**Figure 1:**
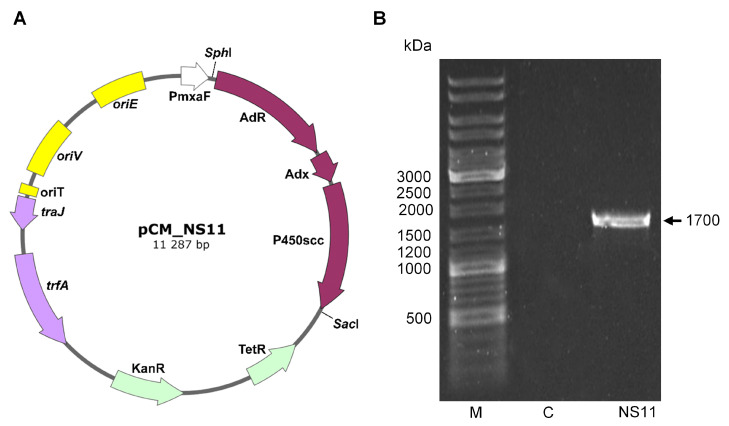
(**A**)—Structure of the shuttle expression vector in *E. coli* and *M. extorquens* strains—plasmid pCM_NS11 containing cDNA encoding bovine AdR, Adx, and cytochrome P450scc (CYP11A1) in a single expression cassette. The plasmid includes the following: P*mxaF*—promoter of methanol dehydrogenase gene; respective origins of replication (oriE) in *E. coli* and (oriV) in *M. extorquens*; *trfA*—the region required for plasmid replication in *M. extorquens*; oriT—origin of conjugative transfer; *traJ*—oriT recognition protein; KanR—kanamycin resistance marker; and TetR—tetracycline resistance marker. (**B**)—Electropherogram of the PCR detection of the Adx_AdR cDNA fragment in *M. extorquens* transformants: M—molecular weight marker; C—negative control *M. extorquens* CM160; and NS11—*M. extorquens* NS11.

The plasmid pNS11 was the source of cDNA encoding enzymes for the initial stage of mammalian steroidogenesis [17]. A PCR fragment of 3365 bp containing the RBS-AdR-RBS-Adx-RBS-CYP11A1 tricistronic cDNA cassette was cloned into the pCM160 vector [29] to form the pCM_NS11 plasmid construct (Figure 1A). The pCM160 and pCM_NS11 plasmids were transferred into *M. extorquens* AM1 cells by conjugation, resulting in the strains *M. extorquens* CM160 and *M. extorquens* NS11.

The presence of the amplicon corresponding to the Adx_AdR cDNA fragment (1.7 kb) confirmed the presence of the desired plasmid construct in the cells of the transformants (Figure 1B).

The *M. extorquens* pCM_NS11 (NS11) strain, expressing the cDNA encoding CYP11A1, AdR, and Adx, under the control of the strong methanol-inducible promoter of the methanol dehydrogenase gene P*mxa*F from *M. extorquens* AM1 and the kanamycin resistance gene as a selective marker (Figure 1A), was obtained and deposited as *M. extorquens* VKM B-3857D. The cloned sequence was confirmed by sequencing.

**Figure 2:**
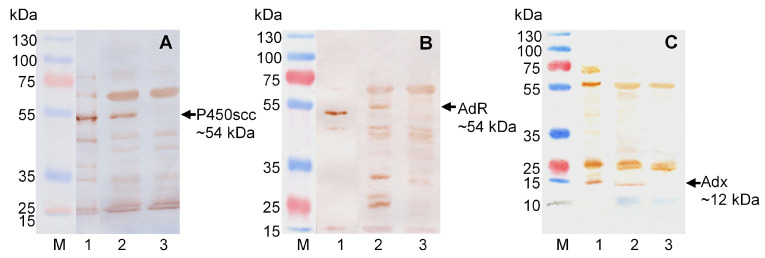
Expression of cholesterol hydroxylase/C20–C22-lyase system in *M. extorquens* cells. SDS-PAGE in 10% (**A**,**B**) or 15% (**C**) gel followed by Western immunoblotting analysis of cell lysates with antibodies against CYP11A1 (**A**), AdR (**B**), or Adx (**C**). 1—lysate of *E. coli* pBar_Triple cells [12] synthesizing mature proteins of the cholesterol hydroxylase/C20-C22-lyase system, 2 µg of total protein (positive control); 2—lysate of *M. extorquens* NS11, 40 µg of total protein; and 3—lysate of *M. extorquens* CM160, 40 µg of total protein (negative control). The arrows indicate the positions of recombinant proteins. Molecular weights of standard marker proteins (kDa) are shown on the left.

Analysis of the bovine cytochrome CYP11A1, Adx, and AdR synthesis in methanol-induced *M. extorquens* NS11 cells, transformed with the pCM_NS11 plasmid, was performed using SDS-PAGE and Western blotting. Samples of the cells carrying pCM160 plasmid were used as a negative control. Forty-eight hours after induction, we harvested cells, prepared the lysates, and performed SDS-PAGE and Western blot analysis using specific antibodies against heterologous proteins (Figure 2). Along with the lysates under consideration, we loaded the lysate of *Escherichia coli* cells transformed with pBar_Triple, directing the synthesis of individual proteins CYP11A1, Adx, and AdR [12] that were used as reference proteins (Figure 2A–C, lane 1). As a result of immunodetection, all three proteins with the expected molecular weights (kDa), P450scc ~54, AdR ~54 kDa, and Adx ~12 (Figure 2A, 2B, and 2C, respectively), were identified in the lysate of the recombinant *M. extorquens* cells. The molecular weight of AdR was slightly higher than that of the native mature protein due to the presence of 6xHis-tag at the N terminus of the protein sequence.

**Figure 3:**
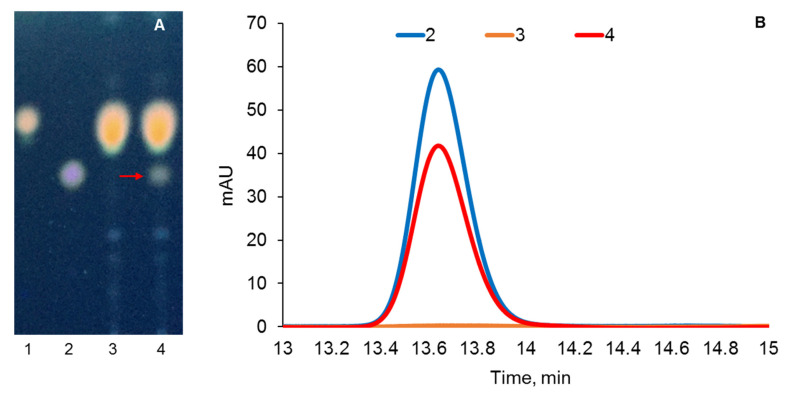
TLC (**A**) and HPLC (**B**) analysis of extracts from culture broths of *M. extorquens* strains on the 11th day of cholesterol bioconversion: 1–cholesterol standard, 2—pregnenolone standard, 3—*M. extorquens* CM160, and 4—*M. extorquens* NS11. Cholesterol (1 g/L) bioconversion was carried out in medium K; methanol (0.5%, *v*/*v*) was added on fourth, sixth, and eight days after inoculation.

The ability of the obtained recombinant strains, *M. extorquens* CM160 and *M. extorquens* NS11, to transform cholesterol was examined in K medium. The presence of pregnenolone in the culture broth on the 11th day of cultivation was detected only in the case of the *M. extorquens* NS11 strain (Figure 3). Pregnenolone production is an unambiguous confirmation of the activity of the steroidogenesis system, namely, AdR, Adx, and CYP11A1 in the *M. extorquens* AM1 strain carrying the pCM_NS11 construct (red arrow and curve, Figure 3).

The structure of obtained pregnenolone was confirmed by MS (C_21_H_32_O_2_ [M + H^+^] = 317.0) and ^1^H and ^13^C-NMR analyses. Spectral data of the product were the following for ^1^H-NMR (400 MHz, DCl3) δ: 5.3 (m, 1H, H-6), 3.5 (m, 1H, H-3α), 2.5 (m, 1H, H-17α), 2.1 (s, 3H, H-21), 1.0 (s, 3H, H-19), and 0.6 (s, 3H, H-18). For 13C-NMR (100.6 MHz, CDCl3) δ, they were as follows: 208.5, 141.0, 121.3, 71.6, 63.7, 57.0, 50.2, 43.9, 42.5, 38.9, 37.3, 36.6, 31.9, 31.9, 31.4, 24.5, 23.0, 21.2, and 13.2.

### 2.2. Visualization of PHB Granules and Cytochrome CYP11A1-GFP in Recombinant M. Extorquens AM1 Cells

As previously shown, the presence of polyhydroxybutyrate (PHB) granules in *B. megaterium* MS941 is important for the bioconversion of cholesterol to pregnenolone by the heterologous cholesterol hydroxylase/C20–C22 lyase system. When the gene encoding eGFP-P450scc fusion protein was expressed in this organism, heterologous proteins were localized in the PHB granules, as suggested by the interaction of membrane domain CYP11A1 with the monolayer phospholipid membrane, which covered PHB granules [18]. This localization of cytochrome CYP11A1 was supposed to facilitate its interaction with the cholesterol substrate, which accumulated in the granules.

*M. extorquens* is known to synthesize PHB [35]. Unlike the PHB granules of *B. megaterium*, the PHB of *M. extorquens* do not have granule-surrounding lipid membranes, but are tightly associated with structural and regulatory proteins, in particular, with low-molecular-weight phasins [36]. In this regard, it was of interest to establish the presence of PHB granules in the cells of recombinant *M. extorquens* and to compare the localization of cytochrome CYP11A1, the main protein of the heterologous cholesterol hydroxylase/C20-C22-lyase system, with PHB granules. For this purpose, studies were performed using fluorescence microscopy. *M. extorquens* AM1 cells were transformed with the constructed plasmid pCM160-P450-GFP containing the cDNA encoding cytochrome CYP11A1 fused to the GFP reporter protein, the use of which allowed for the use of fluorescence detection techniques to determine the localization of the heterologous protein. To visualize PHB granules, the additional staining of recombinant cells with PHB-specific fluorescent dye, Nile red, was performed [37].

**Figure 4:**
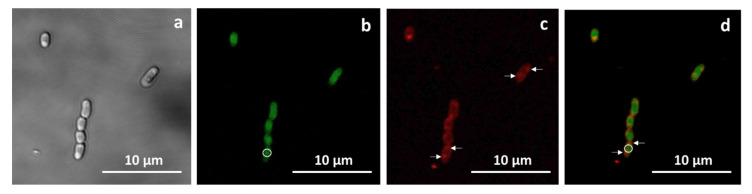
Microphotographs of *M. extorquens* AM1 cells expressing CYP11A1-GFP fusion protein: phase-contrast (**a**), the green emission signal of GFP (**b**), Nile red emission signal (**c**), and overlay, showing overlapping signals in yellow (**d**). Scale bars correspond to 10 μm. White circle indicates CYP11A1-GFP fusion protein distribution, White arrows—PHB granules distribution.

As shown by the analysis of Nile-Red-stained cells using fluorescence microscopy, *M. extorquens* AM1 cells successfully produce PHB granules. Figure 4 shows the localization of PHB granules (Figure 4c,d) and the CYP11A1-GFP fusion protein (Figure 4b,d) in cells. The CYP11A1-GFP fusion protein is distributed throughout the cytosol (Figure 4b,d), with most of it localized closer to the center of cells (Figure 4b, circled), while PHB bodies are located at the edges (Figure 4c, arrowed). Only some overlapping of the green emission signal of GFP with the Nile Red signal can be noted (Figure 4d), but the superposition of these images clearly demonstrates that the distribution of the majority of the heterologous CYP11A1 protein and PHB granules in the recombinant cells differs. Thus, in *M. extorquens* AM1 cells, unlike *B. megaterium*, the cytochrome CYP11A1 does not associate with PHB granules.

### 2.3. Optimization of Conditions for Cholesterol Bioconversion

The comprehensive study included an assessment of the influence of the concentration and regimen of methanol additions, and the effects of nitrogen sources, vitamins, phosphates, and other medium components, as well as the aeration conditions. Taking into account the low aqueous solubility of cholesterol, the effect of the additives of detergents and the application of cyclodextrins as solubilizing agents was investigated.

#### 2.3.1. Medium Composition

As shown in Figure S1, *M. extorquens* strains (recombinant NS11, control CM160, and parental AM1) grew in K and MEM media at almost the same rate, thus indicating that the insertion of the heterologous construct did not affect the culture growth.

As shown in Figure S1, K medium provided higher growth rates compared to growth on MEM medium. However, the growth in K medium was accompanied by significant cell flocculation, and, thus, further experiments were performed using MEM medium, which ensured the more homogeneous growth of methylobacteria.

#### 2.3.2. Regimen of Methanol Addition

**Figure 5:**
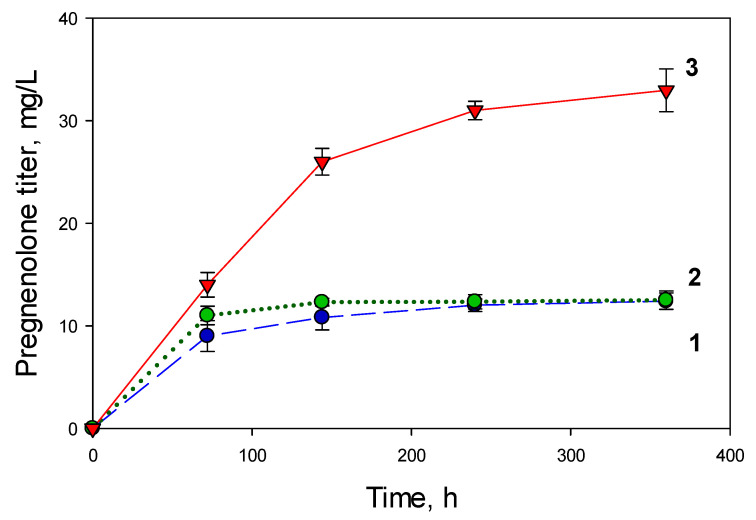
The influence of the methanol concentration and addition regimen on the pregnenolone accumulation by *M. extorquens* NS11: 1 (blue)—mode V1; 2 (green)—mode V2; and 3 (red)—mode V3. Initial cholesterol concentration was 3 g/L.

In the initial experiments, cholesterol (3 g/L) was biotransformed by the recombinant *M. extorquens* NS11 strain at a cholesterol to mCD molar ratio of 1:1.5 in MEM medium with the addition of methanol by portions (0.5 g/L) administered daily following inoculation, or every two days (modes V1 and V2, see Methods). When using these methanol addition modes, weak bioconversion of cholesterol was observed. After three days, the pregnenolone level reached 12.0 ± 0.3 and 12.2 ± 0.7 mg/L, respectively, and did not increase further (Figure 5, curves 1 and 2). Similar data were obtained using K medium with a double addition of 0.5% (vol.) methanol.

To enhance the cholesterol-to-pregnenolone bioconversion, the amount of methanol added was gradually increased during the growth of the culture (V3 mode). Reducing the methanol concentration during the early stages of growth, followed by its subsequent increase, resulted in a 2.75-fold elevation in pregnenolone yield for over ten days of cultivation (Figure 5, curve 3). In subsequent experiments, the methanol addition mode V3 was applied, and the achieved level of pregnenolone accumulation (33.3 ± 2.09 mg/L) was used as the control.

#### 2.3.3. Nitrogen Sources

**Figure 6:**
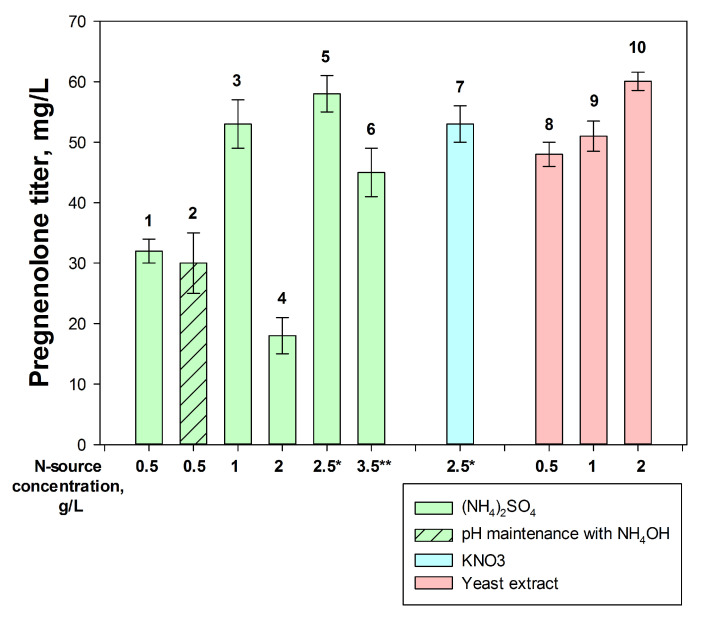
The influence of type and mode of nitrogen sources on pregnenolone accumulation by *M. extorquens* NS11. Final concentration of N-sources is indicated as *X*-axis labels. Variants: 1–6—(NH_4_)_2_SO_4_; 7—KNO_3_; and 8–10—yeast extract. * (NH_4_)_2_SO_4_ or KNO_3_ (0.5 g/L) was added gradually at 0, 2, 4, 6, and 8 days of cultivation; ** (NH_4_)_2_SO_4_ (1 g/L) was added simultaneously with inoculation, and then by portions (0.5 g/L) at 2, 4, 6, 8 days of cultivation. Patterned column—pH maintenance with NH_4_OH. Other columns—pH maintenance with KOH. Initial cholesterol concentration was 3 g/L, the results given for the 11th day of bioconversion.

Ammonium sulfate and potassium nitrate, as well as yeast or corn extracts, were examined as nitrogen sources. As can be seen from Figure 6, the yield of pregnenolone depended on the type, concentration, and addition regimen of nitrogen sources. Thus, an increase in the concentration of ammonium sulfate from 0.5 to 1 g/L led to a more than twofold increase in the yield of pregnenolone (Figure 6, columns 1, 3), while a further elevation in the concentration of ammonium sulfate to 2 g/L resulted in a significant drop in pregnenolone yield (Figure 6, column 4). Notably, when adding ammonium sulfate by 0.5 g/L portions up to a total content of 2.5 g/L (Figure 6, column 5), the maximum pregnenolone titer was achieved. The effect of the portionwise addition of the nitrogen source was also confirmed using KNO_3_ (Figure 6, column 7).

An adjustment in pH level using NH_4_OH (instead of KOH) showed a slight positive effect on cholesterol-to-pregnenolone bioconversion (Figure 6, column 2). A high yield of pregnenolone was observed when a combination of ammonium sulfate (0.5 g/L) and yeast extract (2 g/L) was applied (Figure 6, column 10). Positive, but fewer, effects were observed in the case of the corn extract supplements.

In further experiments, a single addition of 1 g/L (NH_4_)_2_SO_4_ was used as an effective and simple method.

#### 2.3.4. Influence of Other Components

**Figure 7:**
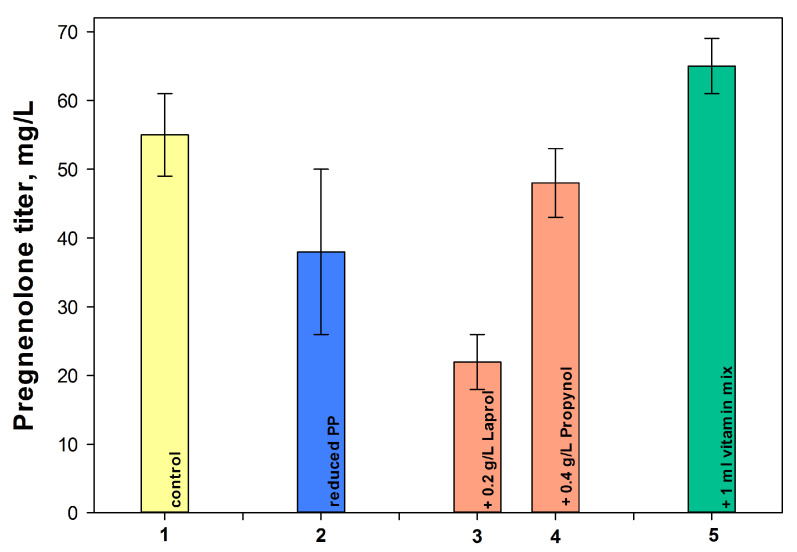
The influence of additional medium components on the pregnenolone production by *M. extorquens* NS11: 1—control (K_2_HPO_4_–2.53 and NaH_2_PO_4_–2.25; 1 g/L (NH_4_)_2_SO_4_), 2—reduced phosphates concentration (0.33 g/L K_2_HPO_4_ and 0.25 g/L NaH_2_PO_4_); 3—0.2 g/L of Laprol; 4—0.4 g/L of Propynol, and 5—1 mL of vitamins mixture. Initial cholesterol concentration was 3 g/L. The data presented is for the 11th day of bioconversion.

The further optimization of the conditions for cholesterol bioconversion by recombinant methylobacteria involved an assessment of reduced phosphate content, and the effects of a vitamin complex and δ-aminolevulinic acid, as well as polymeric antifoam agents. The results are summarized in Figure 7.

As shown in Figure 7, column 2, the reduction in phosphate content in the medium not only did not have a positive effect on the formation of pregnenolone from cholesterol, but also caused large fluctuations in the values, which could, however, be overcome under controlled cultivation conditions in a bioreactor.

The use of antifoams is important for steroid bioconversion. Both antifoams used, Propynol and Laprol, inhibited the bioconversion of cholesterol to pregnenolone, with Laprol having a more significant negative effect, even at its low concentration (0.2 g/L) (Figure 7, columns 3–4).

Many pink-colored facultative methylotrophs are known to be auxotrophs for B vitamins, including pantothenate (vitamin B5) [38]. δ-Aminolevulinic acid is of importance for cytochrome CYP11A1 synthesis, as a precursor of porphyrins [12,39]. Thus, the effect of vitamins and/or δ-aminolevulinic acid on pregnenolone production was studied. Indeed, the addition of the vitamin complex to the medium contributed to an increase in the yield of pregnenolone by 1.15 times compared to the control (Figure 7, column 5). Unexpectedly, the addition of δ-aminolevulinic acid not only failed to stimulate the formation of pregnenolone from cholesterol by the recombinant methylobacteria, but even somewhat reduced the positive effect of adding a vitamin complex

The presence of kanamycin as a selective agent was necessary at the growing stage of recombinant methylobacteria but did not affect the efficiency of cholesterol bioconversion.

#### 2.3.5. The Effect of Surfactants and Detergents

The extremely low aqueous solubility of steroids, and particularly sterols such as cholesterol, in aqueous media is one of the main challenges to their bioconversion. This bottleneck can be partially overcome by using water-miscible detergents and/or surfactants.

We investigated the effects of saponin, a membrane permeabilizer, and Tween 80, a detergent, on the growth and cholesterol bioconversion by the recombinant methylobacterium. The addition of these substances to the bioconversion medium did not affect the growth of bacterial strain, but pregnenolone production was not observed in their presence.

**Figure 8:**
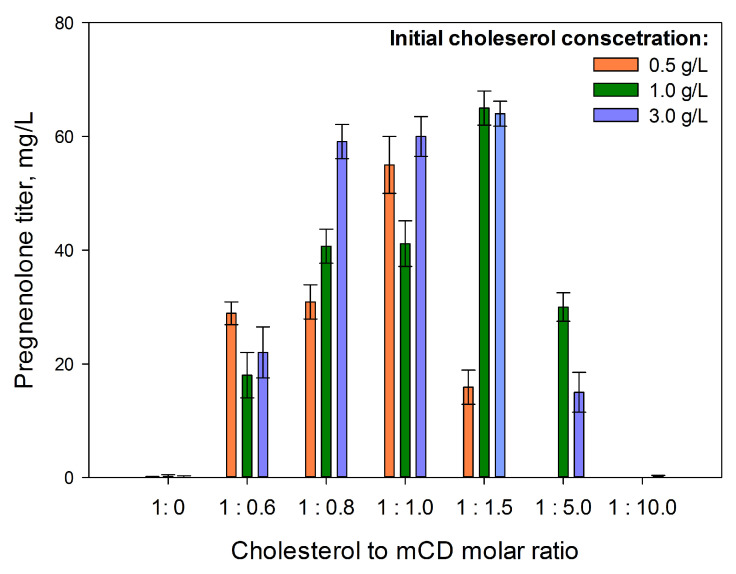
The influence of initial cholesterol concentration (0.5–3 g/L) and its molar ratio to mCD on pregnenolone production by *M. extorquens* NS11. The data presented is for the 11th day of bioconversion.

Methylated cyclodextrins (mCDs) are widely used in steroid bioconversion as effective steroid-solubilizing agents [17,40,41,42,43]. As shown in Figure 8, pregnenolone accumulation during cholesterol bioconversion by *M. extorquens* NS11 depended on both cholesterol and mCD concentrations. The maximum pregnenolone yield was observed when using cholesterol (1–3 g/L) at a molar ratio of 1.5:1 to mCD, whereas a further increase in mCD content resulted in a significant decrease in pregnenolone yield.

It is worth noting that the maximum obtained titer of pregnenolone remains virtually unchanged with a 2- and 6-fold increase in substrate concentration, apparently due to the limitation of cholesterol transport through the membrane of methylobacteria. The portionwise addition of mCD, starting from the second day of culture growth in the presence of cholesterol, as well as the gradual increase in its concentration according to the plan described in Section 4 ‘Materials and methods’, led to a decrease in the titer of pregnenolone (Figure S2).

The cyclodextrin molecules are characterized by the hydrophilic surface and a hydrophobic cavity in which a steroid molecule can be entrapped [44], and the formation of the steroid–cyclodextrin inclusion complex may facilitate the steroid bioconversion [42,45]. The CD-mediated effects on steroid transport through the cell envelopes and the up-regulation of protein synthesis have been described as contributing to the enhancement of steroid bioconversion [41,46].

An increase in the bioconversion efficiency in the presence of mCD was shown with regard to sterol transformation using actinobacteria [42,47], yeasts [48], and *E. coli* carrying genes of the CHL system resting cells [13]. In the latter case, the maximum pregnenolone yield was recorded at a molar ratio of 1:2.8 of cholesterol to mCD.

Nonetheless, mCDs in high concentrations can negatively affect the steroid bioconversions [42,48].

#### 2.3.6. The Influence of Culture Density and Final Optimization

**Figure 9:**
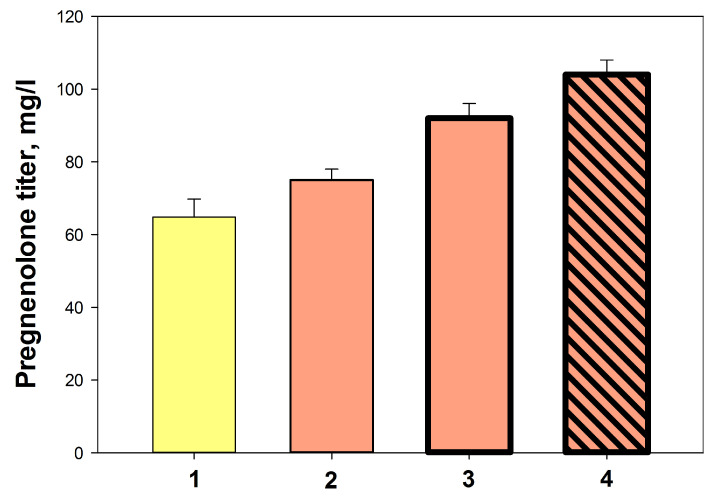
The influence of biocatalyst (*M. extorquens* NS11) cell density and transformation conditions optimization on pregnenolone accumulation: 1—control (10% of inoculum); 2, 3, and 4—1.5 g/L of concentrated washed wet biomass was used: 3 and 4—(NH_4_)_2_SO_4_ concentration in transformation media was evaluated from 1 to 3 g/L; and 4—gas-to-liquid volumes ratio was enlarged from 6.5 to 14 (or gas phase volume from 650 to 700 mL).

As noted above, the use of MEM medium ensured the homogeneous and stable growth of the recombinant *M. extorquens* NS11 but did not allow a dense culture to be obtained. The use of concentrated wet biomass (Section 4, Materials and Methods) resulted in a slight increase in pregnenolone yield (Figure 9, 2).

A more significant effect was observed when adding ammonium sulfate to the bioconversion medium and changing the aeration conditions (Figure 9, variants 3, 4). The maximum pregnenolone yield (103.8 ± 2.7) in mg/L was achieved by using concentrated biomass, adding ammonium sulfate to the medium, and increasing aeration (Figure 9, 4).

## 3. Discussion

The heterologous expression of eukaryotic steroidogenesis systems in microbial hosts opens up prospects for the one-stage biotechnological production of valuable steroid compounds from natural sterols. Gram-positive bacteria of the genus *Mycolicibacterium* are most often used as expression chassis in such studies. These actinobacteria have effective systems for transporting lipophilic compounds, including steroids. The advantages of their use also include a relatively high growth rate. However, the presence of their own sterol catabolism systems can significantly complicate the functioning of heterologous steroidogenesis proteins when using actinobacteria as an expression chassis. Thus, the presence of high 3-hydroxysteroid dehydrogenase activity (3-HSD) in actinobacteria leads to the oxidation of cholesterol to become cholestenone, which is not a substrate for eukaryotic cytochromes [49]. To solve this problem, attempts were made to knock out the genes encoding cholesterol oxidase and 3-hydroxysteroid dehydrogenase; however, the complete suppression of 3-HSD activity was not achieved (e.g., [50]). In addition, cytochromes P450, accounting for initial reactions of the sterol side chain degradation in mycolicibacteria (e.g., CYP 125 or CYP142), may compete with heterologous CYP11A1 for the sterol substrate.

In this study, we explored the following alternative approach: using a microorganism lacking its own steroid oxidation system as an expression chassis. The pink-colored Gram-negative methylobacterium, *Methylorubrum extorquens*, was chosen as such a micro-organism.

Indeed, the strain expressing genes encoding the initial stage of steroidogenesis of the bovine adrenal cortex, as part of the tricistronic operon (CYP11A1-Adx-AdR) obtained in this work, oxidized cholesterol with the formation of pregnenolone as the only product. Notably, in recombinant *M. extorquens* AM1 (pNS11) cells, heterologous cytochrome CYP11A1 is not associated with the surface of PHB-formed granules, in contrast to what was shown previously for recombinant *B. megaterium* [18].

**Figure 10:**
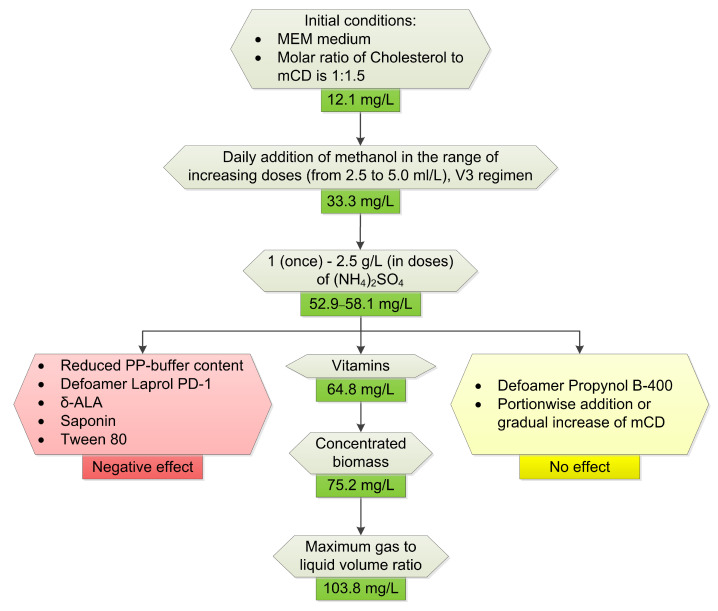
General scheme of the optimization of cholesterol bioconversion to pregnenolone using *M. extorquens* NS11.

The presence of a heterologous steroidogenesis system in *M. extorquens* NS11 did not affect the growth characteristics of the strain. To increase the yield of pregnenolone, the conditions for cholesterol bioconversion were optimized (Figure 10).

Key factors influencing the efficiency of the cholesterol bioconversion included using a dropwise methanol addition (Figure 5), a nitrogen source (Figure 6) and vitamins (Figure 7), the application of mCD (Figure 8) as a solubilizing agent, a concentrated biomass, and intensive aeration (Figure 9). As a result, the yield of pregnenolone increased almost 4-fold (from 28.3 to 103.8 mg/L, Figure 10).

It is important to note that pregnenolone was obtained as the only product of cholesterol oxidation and was not further metabolized. The absence of product degradation, characteristic of mycolicibacteria as host organisms, eliminates the need for substrate modification and product regeneration steps, and simplifies the isolation and purification of the target product, thereby reducing the overall cost of the process. This advantage makes whole-cell catalysts based on methylobacteria promising, in contrast to the mycolicibacteria previously used for these purposes.

In conclusion, the results obtained demonstrate the possibility of using methylobacteria that lack their own steroid degradation systems to reconstruct the bovine cortex cytochrome CYP11A1 enzymatic system, and indicate the prospects for their use in creating biocatalytic strains including other eukaryotic steroidogenic P450 systems.

## 4. Materials and Methods

### 4.1. Reagents

The following materials and reagents were used in the work: agar-agar, tryptone, corn and yeast extracts (Panreac, Barcelona, Spain), ethidium bromide, sodium dodecyl sulfate (Serva, Heidelberg, Germany), EDTA, Tris-HCl, (Bio-Rad, Hercules, CA, USA), agarose (Invitrogen, Paisley, UK), cholesterol, pregnenolone (Serva, Germany; randomly methylated β-cyclodextrin (RAMEB) W7 M1.8 (Wacker Chemie, Munich, Germany), horseradish peroxidase, diaminobenzidine tetrahydrochloride hydrate, Nile red (Sigma-Aldrich, St. Louis, MO, USA), DNA molecular weight markers “GeneRuler DNA Ladder Mix”, and proteins molecular weight markers «Color Pestained Protein Standard» (New England Biolabs, Ipswich, MA, USA). Primary rabbit polyclonal antibodies (IgG fraction) against bovine CYP11A1 (P450scc), AdR, and Adx were kindly provided by Prof. Shkumatov (Institute of Physico-Chemical Problems, Minsk State University, Minsk, Belarus). All other reagents were purchased from domestic suppliers (Russia) and were of chemical purity grade.

The isolation and purification of DNA was carried out using kits “QIAprep Spin Miniprep Kit” (QIAGEN, Germantown, MD, USA), “NucleoSpin Plasmid”, and “NucleoSpin Extract II” (Macherey-Nagel, Duren, Germany). DNA modifications were performed by restriction endonucleases and T4 DNA ligase (Thermo Scientific, Waltham, MA, USA). All DNA manipulations were performed in accordance with manufacturer’s recommendations.

### 4.2. Strains and Media

The *M. extorquens* AM1 (CIP 106,787 = DSM 6343 = VKM B-2064T) parental strain was used as a chassis for genetic manipulations. To obtain seed cultures and conduct the cholesterol bioconversion by parental or recombinant strains of *M. extorquens*, “K” medium [51] and the constructed “MEM” medium were used, which were prepared in the form of two solutions, sterilized separately, solution A (g/0.9 L: K_2_HPO_4_—2.53, NaH_2_PO_4_–2.25, (NH_4_)_2_SO_4_—0.5, pH 7–7.2) and solution B (g/0.1 L: MgCl_2_ × 6H_2_O—0.1, CaCl_2_—0.002, ZnSO_4_—0.00034, MnCl_2_—0.0002, FeSO_4_—0.005, (NH_4_)_6_Mo_7_O_24_ × 4H_2_O—0.0025, CuSO_4_—0.00025, CoCl_2_—0.0005). After autoclaving the medium, methanol, previously sterilized by filtration, was added. The culture was grown aerobically at 29 °C and with shaking at 200 rpm. Agarized media were prepared by adding 15 g/L agar-agar to solution A.

*Escherichia coli* strains DH5α and S17-1, used for replication and the conjugation transfer of plasmids, were grown in LB medium [52]. Kanamycin (50 mg/L) was used to select and maintain recombinant strains.

### 4.3. Construction of Plasmids and Recombinant Strains

The gene cassette encoding RBS-AdR-RBS-Adx-RBS-CYP11A1 was PCR-amplified, using NS11for and NS11rev primers containing flanking *Sph*I and *Sac*I restriction sites from the pNS11 plasmid (Table 1). The cDNA sequence encoding the CYP11A1-GFP fusion protein was obtained by PCR amplification using the pCoxIV-CHL-GFP plasmid (Table 1), forward, and reverse primers containing *Sph*I and *Sac*I restriction sites—GFPfor and GFPrev (Table 1), respectively. The PCR products were digested with *Sph*I/*Sac*I and inserted into the pCM160 vector treated with the same restriction enzymes. The resulting target plasmids, pCM_NS11 (Figure 1A) and pCM160-P450-GFP, contained cDNA encoding AdR-Adx-CYP11A1 and the CYP11A1-GFP fusion protein under the control of the P*mxaF* promoter, respectively. The structure of the plasmids was confirmed by restriction analysis and sequencing.

The plasmids were transferred into competent *E. coli* BL21(DE3) cells by heat shock. For conjugation transfer, *M. extorquens* AM1 culture, at the early exponential growth phase (OD_600_ = 0.2–0.3), was mixed with an overnight culture of transformed *E. coli* S17-1 cells, previously washed with an equivalent volume of K medium. The ratio of donor *E. coli* S17-1 cells to recipient *M. extorquens* AM1 cells was 1:1, in accordance with OD_600_. The cell mixture concentrated on a membrane filter (0.45 μm, Millipore, Merck, Darmstadt, Germany) was placed on the agar K medium, containing 0.02% casamino acids and 0.5% methanol, and was incubated at 29 °C for 24 h. The filters were washed with 2 mL of K medium. The cell suspension was plated on selective K medium containing 50 μg/mL kanamycin and 0.5% methanol as a sole source of carbon and energy. The grown pink colonies of *M. extorquens* transconjugants were selected for further work. The result of the plasmid transfer was assessed by PCR, using the total DNA of cells of individual colonies of the *M. extorquens* NS11 as a template, and a pair of detection primers Adrf/Adxr. The biological objects used and created in this work are presented in Table 1.

### 4.4. Gene Expression and Protein Analysis

*M. extorquens* NS11 cells were grown in MEM medium with 5 mL/L methanol for 48 h, as described in Materials and Methods, Section 4. Cell lysates were analyzed using the SDS-PAGE technique [55] followed by Western blotting [56]. To obtain the lysates of cells, microbial cells were pelleted by the centrifugation of 3.0 mL of culture at 5000 rpm for 10 min, and then the pellet was resuspended in a buffer to prepare the samples for electrophoresis (sample buffer) and disrupted by boiling (2 min, 100 °C). Protein content was measured according to Lowry et al. (1951) [57]. Then, 5–70 µg of total protein was loaded into each lane. Proteins separated by SDS-PAGE in 10–15% gels were transferred to Hybond^TM^-C Extra nitrocellulose membrane (Amersham Biosciences, Buckinghamshire, UK) using semidry blotting. The expression of heterologous proteins was detected using the primary antibodies against P450scc (6.0 mg/mL), AdR (9.5 mg/mL), or Adx (2.5 mg/mL) at 1:7500, 1:7500, and 1:4500 dilution (*v*/*v*), respectively, conjugate of anti-rabbit-IgG antibodies with horseradish peroxidase (Sigma-Aldrich, St. Louis, MO, USA, 1 mg/mL) at 1:15,000 dilution (*v*/*v*) and the peroxidase substrate diaminobenzidine tetrahydrochloride hydrate (DAB, Sigma-Aldrich, St. Louis, MO, USA) (0.25 mg/mL). Primary rabbit polyclonal antibodies (IgG fraction) against bovine P450scc, AdR, and Adx were kindly provided by Prof. V.M. Shkumatov (Institute of Physico-Chemical Problems, Minsk State University, Minsk, Belarus).

### 4.5. M. Extorquens AM1 Cells Staining and Fluorescence Microscopy

A dye solution (mixture of 3 μL 2 mM Nile Red (NR) and 2.5 μL 2 mM MCD) was added to the aliquot (0.5 mL) of the methanol-induced *M. extorquens* AM1 harboring the pCM160-P450-GFP cell culture, and the sample was incubated with shaking for 40 min at room temperature. Then the cells were pelleted (8000 rpm, 5 min), washed two times with mixture of phosphate-buffered saline (PBS) and MCD (final concentration 48 μM), and fixed with 1 mL of 3% formaldehyde in PBS for 20 min at room temperature. The fixed cells were spun down, washed once with PBS, suspended in 10 μL of PBS, and used for the preparation of slides. Cell fluorescence was analyzed under a fluorescent microscope Eclipse Ts100 (Nikon, Tokyo, Japan) in a confocal mode using the following excitation–emission maxima: 530/650 nm for Nile red and 395/507 nm for GFP.

### 4.6. Bioconversion of Cholesterol

The seed culture of recombinant strains was obtained in two stages, in K or MEM medium supplemented with 50 mg/L kanamycin and 5 mL/L methanol. The first inoculum was grown in 5 mL of the above media in 40 mL tubes at 30 °C and 180 rpm, for 48 h. The second inoculum was grown in 100 mL of the media with 5% (vol.) of the first inoculum in 750 mL flasks at 29 °C and 200 rpm for 48 h.

Cholesterol (0.5–3 g/L) was added to MEM medium as a mixture with methyl-β-cyclodextrin (mCD) (molar ratio of cholesterol to mCD varied in a range of 1:0.6 to 1:5). The initial cholesterol-to-mCD molar ratio was 1:1.5 at the cholesterol load of 3 g/L (shown in Figure 5, Figure 6 and Figure 7). Bioconversion was carried out in 750 mL flasks containing 80 mL of fresh media with 20 mL of second inoculum (20% vol.) at 29 °C and 200 rpm for 240 h. The pH of the culture broth was maintained in the range of 6.8–7.2 by sterile 10% solutions of NaOH and H_2_SO_4_ if necessary.

### 4.7. Optimization of Bioconversion Conditions

***Medium composition.*** The composition of the media was optimized using single-factor and multi-factor approaches. The influence of carbon (methanol) and nitrogen (ammonium sulfate or potassium nitrate) sources was studied by varying their initial concentrations in the nutrient medium or changing the mode of their addition.

Three methanol addition regimens were investigated:

V1—5 mL/L daily since inoculation;

V2—5 mL/L since inoculation and then every two days;

V3—daily: 2.5 mL/L since the inoculation daily until the third day; 3.75 mL/L daily from the fourth to the sixth day; and 5 mL/L daily from the seventh to the ninth day.

The influence of organic (yeast and corn extracts) and inorganic nitrogen (ammonium or nitrate salts) sources was assessed. The initial concertation of (NH_4_)_2_SO_4_ or KNO_3_ in the bioconversion media was 0.5 g/L. The effect of the amount of nitrogen sources and their fractional adding during the cholesterol bioconversion process was assessed. An approach in which the culture liquid was titrated with 10% NH_4_OH was also investigated.

The effect of the addition of the following components to the MEM medium was studied: 1 mmol/L δ-aminolevulinic acid (δ-ALA); 0.25 or 1 mL/L of vitamins solution (g/L methanol: biotin—0.02, p-aminobenzoic acid—0.3, nicotinic acid—0.2, lipoic acid—0.05, calcium panthetonate—0.1, pyridoxine dihydrochloride—0.5, folic acid—0.05, cyanocobalamin (B12)—0.1, thiamine—0.2, and riboflavin—0.1); and 0.2 or 0.4 g/L of defoamers Laprol PD-1 (CAS № 25322-68-3) or Propynol B-400 (CAS № 60617-03-0). In some experiments, phosphate’s concentration was reduced to 0.33 g/L K_2_HPO_4_ and 0.25 g/L NaH_2_PO_4_, if specified.

***Cholesterol addition*.** Taking into consideration the extremely low aqueous solubility of cholesterol, to improve its availability for the biocatalytic system, cholesterol was introduced to the media in the mixture with mCD (randomly methylated β-cyclodextrin or RAMEB), 1 g/L saponin, or 0.1 g/L polysorbate 80 (Tween 80), as well as their combinations if mentioned. Cholesterol–mCD mixture was added before autoclaving at the molar ratios of 1:0.6, 1:0.8, 1:1, 1:1.5 (control variant), 1:5, or 1:10, or without any mCD addition. Otherwise, mCD was added fractionally during cultivation, as follows:

Once: at the second day—15.3 g/L (molar ratio to cholesterol is 1.5:1);

Twice: at the first and second day—15.3 g/L, 30.6 g/L in total (molar ratio of mCD to cholesterol is 3:1);

Twice: at the first and fourth day—15.3 g/L, 30.6 g/L in total (molar ratio of mCD to cholesterol is 3:1);

Twice: at the first —15.3 g/L—and at the second day—30.6 g/L—with 45.9 g/L in total (molar ratio of mCD to cholesterol is 4.5:1);

Three times: at the first, second and fourth days—15.3 g/L—with 45.9 g/L in total (molar ratio of mCD to cholesterol is 4.5:1).

***The influence of seed culture density.*** The concentrated culture was obtained by centrifugation, at 20 °C and 4000× *g* for 10 min, of the strain *M. extorquens* NS11, grown for 24 h. The precipitated cells were resuspended in sterile tap water and added to 750 mL flasks containing 50 or 100 mL of bioconversion medium at a concentration of 1.40–1.65 g/L. So, the ratio of liquid-to-gas volumes (indirectly the effect of aeration) on the bioconversion of cholesterol into pregnenolone was studied in 750 mL flasks containing 50 or 100 mL of MEM medium, i.e., the obtained ratios were 1:14 and 1:6.5.

### 4.8. Steroids Assay

Steroids assay was performed by High-Performance Liquid Chromatography (HPLC), Thin Layer Chromatography (TLC), Mass-Spectrometry (MS), and Nuclear Magnetic Resonance (1H-NMR and 13C-NMR Spectroscopy).

Steroids were extracted from 0.25 mL of culture broth by 10× volume of acetonitrile, then centrifuged for 10 min at 12,100× *g* (Eppendorf, Enfield, CT, USA). Supernatant was analyzed using Agilent 1200 (Agilent Technologies, Waldbronn, Germany) equipped with Symmetry C18 column (4.6 × 250 mm, 5 μm) and pre-column Symmetry C18 (3.9 × 20 mm, 5 μm) (Waters, Milford, MA, USA), at 50 °C and a flow rate of 1 mL/min. The mobile phase was acetonitrile: water: methyl tert-butyl ether: trifluoroacetic acid (40:60:8:0.02, *v*/*v*). Steroids were detected at 200 nm. Calibrations were performed by the external standard method based on peak areas. Retention time (Rt) for pregnenolone was 13.65 min. The results were processed using ChemStation Rev. B.04.03 (Agilent Technologies, Santa Clara, CA, USA).

The cultivation broth was extracted with double volume of ethyl acetate and applied on TLC plates (ALUGRAM SIL G/UV254, Marchery-Nagel, Duren, Germany), which were developed in benzene–acetone mixture (7:1, *v*/*v*). After drying, the plates were sprayed with MnCl_2_ solution (0.18 g MnCl_2_ dissolved in 100 mL water–ethanol mixture (1:1, *v*/*v*) acidified with 3 mL H_2_SO_4_), heated at 105 °C for 5–10 min, and visualized under UV light (365 nm) using a hemiscope (CN-15MC UV Darkroom, Vilber Lourmat, Collegien, France).

MS, 1H-NMR, and 13C-NMR spectroscopy were performed as described in [17].

### 4.9. Statistical Analysis 

All the experiments were performed three to five times and the data were statistically analyzed by one-way ANOVA. Standard deviation is shown as error bars at figures.

## Figures and Tables

**Table 1:** Bacterial strains, plasmids, and DNA primers used in the study.

Strain or Plasmid	Description	Source or Reference
Plasmids:		
pNS11	The pMyNT vector included 3.3 kb fragment with cDNA, encoding RBS-AdR-RBS-Adx-RBS-CYP11A1	[17]
pCM160	*E. coli* и *M. extorquens* shuttle expression vector, Km^R^, P*mxaF*, ColE1-IncP	[29]
pCM_NS11	pCM160 vector included 3352 bp fragment with cDNA, encoding RBS-AdR-RBS-Adx-RBS-CYP11A1 inserted by sites SacI-SphI	Current research
pCoxIV-CHL-GFP	Plasmid containing cDNA for GFP-fused CHL polyprotein	[53]
pCM160-P450-GFP	Plasmid includes cDNA for CYP11A1-GFP inserted in the frame with lacZa sequence together	Current research
Strains:		
*Escherichia coli* DH5α	F^–^ φ80*lac*ZΔM15 Δ(*lac*ZYA-*arg*F)U169 *rec*A1 *end*A1 *hsd*R17(r_K_^−^, m_K_^+^) *pho*A *sup*E44 λ^–^*thi*-1 *gyr*A96 *rel*A1	Thermo Fisher Scientific, USA
*Escherichia coli* S17-1	RP4-2(Km::Tn7,Tc::Mu-1), pro-82, LAMpir, recA1, endA1, thiE1, hsdR17, creC510	[54]
*Methylorubrum extorquens* AM1	Model strain used to study methylotrophy and biotechnological applications of methylobacteria	Deposited in all-Russian collection of microorganisms (IBPM) as VKM B-2064T
Primers:		
NS11for	CACTAA**GCATGC**GCCACCACCCGATAAGAG *	Current research
NS11rev	A**GAGCTC**GCTATCGATAAGCTTTCA *	Current research
Adrf	ATGGCGAGCACTCAAGAACAAAC	Current research
Adxr	ATCTGGCAGCCCAACCGCGATC	Current research
GFPfor	GCTCCAGATATC**GCATGC**TGTCCACAAAGACCC *	Current research
GFPrev	CAGC**GAGCTC**AATTCATTATTTGTACAGCTCATCC *	Current research

* *Sph*I and *Sac*I restriction sites are in bold.

## Data Availability

The original contributions presented in this study are included in the article/Supplementary Material. Further inquiries can be directed to the corresponding authors.

## Supplementary Materials

The following supporting information can be downloaded at: https://www.mdpi.com/article/10.3390/ijms262210975/s1.
