# Supplementary material for: Pregnenolone Bioproduction in Engineered Methylobacteria: Design and Elaboration
Source: Int J Mol Sci. 2025 Nov 13;26(22):10975. doi: 10.3390/ijms262210975 (PMC12652760; doi:10.3390/ijms262210975)
Supplement: Supplementary file 1 [file ijms-26-10975-s001.zip › Figure S1.pdf]

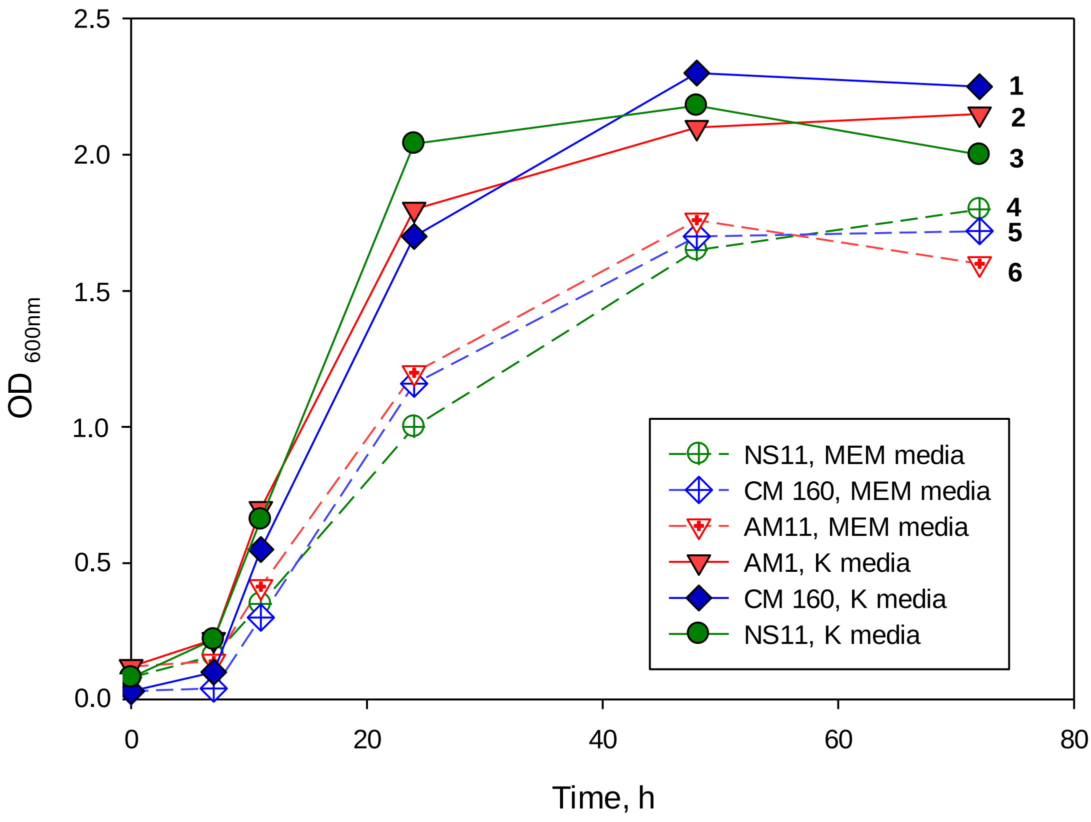

Figure S1. *M. extorquens* strains growth curves in K medium (1-3) and in MEM medium (4-6): 3, 4 – *M. extorquens* NS11; 1, 5 – *M. extorquens* CM 160; 2, 6 – *M. extorquens* AM1 (parental strain). The experiments were carried out three times, the experimental error was calculated the standard deviation.
