# Supplementary material for: Pregnenolone Bioproduction in Engineered Methylobacteria: Design and Elaboration
Source: Int J Mol Sci. 2025 Nov 13;26(22):10975. doi: 10.3390/ijms262210975 (PMC12652760; doi:10.3390/ijms262210975)
Supplement: Supplementary file 1 [file ijms-26-10975-s001.zip › Figure S2.pdf]

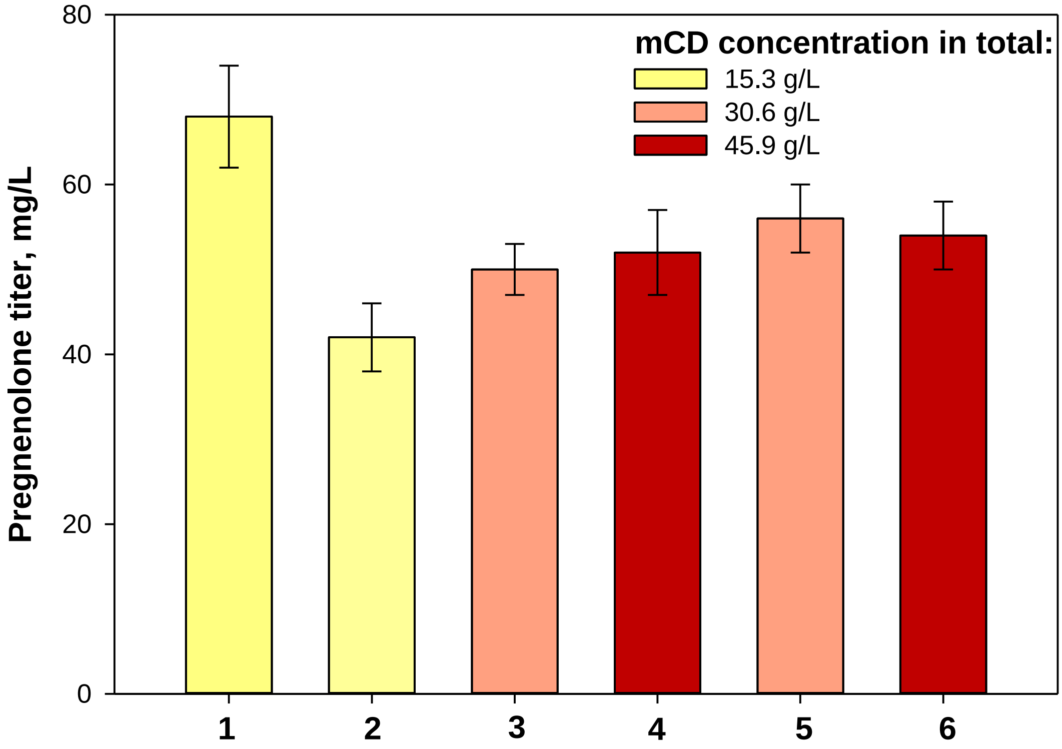

Figure S2. The influence of mCD addition regimen on pregnenolone production by *M. extorquens* NS11: 1 – control (the addition of mCD at the 1st day of bioconversion, molar ratio - 1:1.5, 15.3 g/L mCD), 2 – the addition on the 2nd day of growth (molar ratio - 1:1.5, 15.3 g/L mCD); 3 – the addition on the 1st and 2nd days (total molar ratio – 1:3, 30.6 g/L mCD); 4 - the addition on the 1st, 2nd and 4th days (total molar ratio - 1:4.5, 45.9 g/L mCD); 5 – the addition on the 1st and 4nd days (total molar ratio – 1:3, 30.6 g/L mCD); 6 – the addition on the 1st and 4th days double dose (molar ratio – 1:4.5, 45.9 g/L mCD. The initial cholesterol concentration was 3 g/L, the data presented for 11th day of bioconversion.
